# The Impact of Landscape Complexity on Invertebrate Diversity in Edges and Fields in an Agricultural Area

**DOI:** 10.3390/insects7010007

**Published:** 2016-02-03

**Authors:** Tracy R. Evans, Meredith J. Mahoney, Everett D. Cashatt, Jinze Noordijk, Geert de Snoo, C. J. M. Musters

**Affiliations:** 1 Institute of Environmental Sciences, Leiden University, Einsteinweg 2, Leiden 2333 CC, The Netherlands; snoo@cml.leidenuniv.nl (G.S.); musters@cml.leidenuniv.nl (C.J.M.M.); 2 Illinois State Museum Research and Collections Center, 1011 E. Ash Street, Springfield, IL 62703, USA; mjmahoney@museum.state.il.us (M.J.M.); cashatt@museum.state.il.us (E.D.C.); 3 European Invertebrate Survey (EIS)/Naturalis Biodiversity Center, PO Box 9517, Leiden 2300 RA, The Netherlands; jinze.noordijk@naturalis.nl

**Keywords:** biodiversity, taxonomic richness, diversity index, landscape complexity, North American agriculture

## Abstract

Invertebrate diversity is important for a multitude of ecosystem services and as a component of the larger ecological food web. A better understanding of the factors influencing invertebrate taxonomic richness and diversity at both local and landscape scales is important for conserving biodiversity within the agricultural landscape. The aim of this study was to determine if invertebrate richness and diversity in agricultural field interiors and edges in central Illinois, USA, were related to the complexity of the surrounding landscape. Our results show taxonomic richness and diversity in field edges is positively related to large scale landscape complexity, but the relationship is negative for field interiors. These unexpected results need further study.

## 1. Introduction

Agriculture is intensifying to meet the growing demand for food for the increasing numbers of people and livestock. Fields have increased in area resulting in the loss of non-crop field margins [1,2,3,4]. Chemical usage has increased, harvesting technologies have improved, and tillage frequency has increased. There is a known negative relationship between agricultural intensity and biodiversity [5,6,7].

There are numerous benefits to conserving or restoring biodiversity in agricultural areas including provision of habitat for highly valued farmland birds [6,8,9], game species [10] and economically relevant species of invertebrates [11]. Habitat conservation and restoration support ecosystem services such as pollination [12], erosion control [13] and natural pest control [6,11,14]. These practices enhance floral diversity within crops [15] and serve as corridors to link protected areas for various species [16,17]. Invertebrates provide ecosystem services such as pollination and pest control, although some species are agricultural pests [18,19]. Habitat restoration in the USA is largely focused on large blocks of land. However, small narrow landscape elements like field edges, road sides, and ditch and creek banks may also play an important role in agricultural landscapes [20]. Being (literally) marginal, management of these vegetated elements to maximize biodiversity would not diminish agricultural production.

Previous work has shown that both local and landscape factors affect the biodiversity of semi-natural elements in agricultural areas and that the effectiveness of management for biodiversity depends on the landscape complexity [2,3]. Agricultural landscapes may be categorized as complex, simple or cleared [3]. In general, as the complexity of the landscape increases, biodiversity increases, although some species groups are insensitive to it [3,21].

Much research has been focused on typical European agricultural landscapes [2,6,22] but other than utilization as corridors [23], there has been little research on the biodiversity of agricultural field edges (area next to cultivated fields) in the USA. In this paper, we studied invertebrate diversity in and around agricultural fields in Illinois, Midwest USA. In this area, tall grass prairie has been largely displaced by intensive agriculture, making it important to conserve and restore remaining biodiversity. In addition, as financial incentives are provided to farmers to adopt environmentally friendly agricultural practices and take some tracts out of agricultural production [24], there is a need to study how and where biodiversity is best promoted to ensure funding is spent optimally. There is little baseline data on the relative importance of landscape factors affecting invertebrate taxonomic richness (TR) and diversity index (DI) in Midwestern field edges. [25,26]. We think that management of the narrow elements (edges outside tilled area of fields) can be modified to enhance invertebrate diversity conservation and restoration in the Midwest. Invertebrate diversity in non-cultivated field edges (FE) might influence the richness and diversity of invertebrates in the cultivated field interiors (FI), which is important information for farmers given the ecosystem services these animals might provide [27]. We examined invertebrate diversity in and adjacent to 30 agricultural fields in three counties in central Illinois. Our central hypotheses were that, first, FE have a higher TR and DI than FI and, second, in both FE and FI, TR and DI would be greater as landscape complexity increased due to a larger regional species pool [28]. We also examined local factors such as vegetative structure that could have affected TR and DI independently of landscape complexity.

## 2. Experimental Section

### 2.1. Study Area

**Figure 1:**
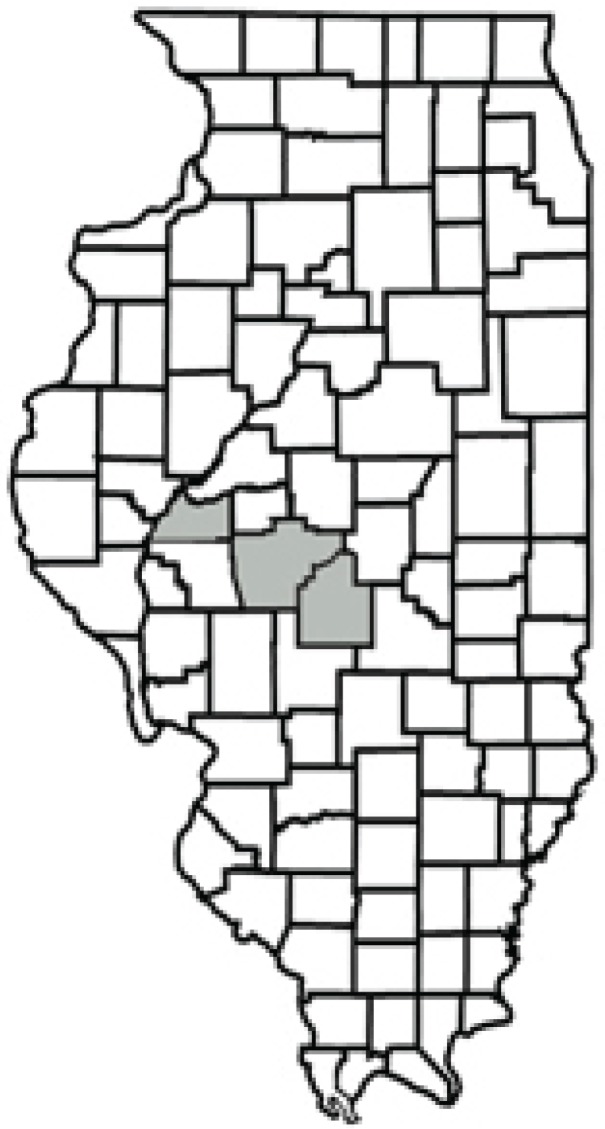
Location of Cass, Sangamon and Christian counties in Illinois, IL, USA.

The study was conducted in 2011 and 2012 in central Illinois in Cass, Christian and Sangamon counties (Figure 1). This is part of the Grand Prairie Natural Division, a vast plain of formerly tall grass prairie [29]. Soils are fertile and developed from glacial outwash, lakebed sediments and deposited loess. Natural drainage is poor but farmland drainage has been improved with the use of tile lines and ditches. The topography is generally level to rolling. Illinois climate is typically continental with cold winter temperatures (mean −3.8 °C), warm summers (24.6 °C) and frequently fluctuating temperature, humidity, cloud cover and wind conditions. Precipitation averages 1006 mm per annum and the growing season is ~185 days. In 2012, precipitation was much below normal (595.12 mm) (Midwestern Regional Climate Center, Springfield, Illinois: http://mrcc.isws.illinois.edu/CLIMATE). Due to the reduced precipitation and high ambient temperatures, the region was considered to be in an extreme drought (National Oceanic and Atmospheric Administration 2012).

We selected ten agricultural fields mostly seeded in a 2–3 year corn (*Zea mays*) and soybean (*Glycine max*) planting rotation in each of three counties for a total of 30 fields (Supporting Information, Table S1). Fields were visually selected for varied edge structure. The average field size was 28 ha with a range from 1–117 ha (Table S2). Fields differed in their surrounding structural complexity, ranging from simple landscapes with a relatively high percentage of arable land, to complex landscapes with a relatively low percentage of arable land and a large proportion of semi-natural land cover and other land use types (Table S3). The edge structure and vegetation ranged from closely mown grass monoculture to shrubby vegetation more than a meter in height (Table S2). Permission to access the fields was obtained from land managers and landowners (in many cases the landowner was different from the land manager). Vegetation in the FE was managed by various entities including the landowner, land manager, and township employees and consisted of a variety of mowing and herbicide regimes. Prior to the start of the study, FIs had been seeded with genetically modified (Roundup Ready) corn or soybeans by the landowners or managers (Table S1). Roundup Ready seeds are modified to be resistant to glyphosate type herbicides that are used to control weeds.

ArcView GIS 3.2 and ArcGIS Spatial Analyst (Environmental Systems Research Institute, Inc., Redlands, CA, USA) were used to determine field area (ha), field edge length (m), width of FE (m), distance to nearest large non-agricultural area (>1 ha), proportion of non-agricultural area at three different scales, and soil type. Field length was the length of the field adjacent to the FE where traps were placed. Complexity, *i.e.*, proportion of non-agricultural area to agriculture, was determined using nested circular buffers (with radii of 500 m, 1000 m and 6000 m) around the center of each group of samples per field. We used existing landcover classifications from satellite imagery [30]. We defined agriculture as arable land sown in corn, soybean, winter wheat (*Triticum aestivum*) or other row crops. We defined non-agricultural areas as those classified as upland forest, savannah, coniferous forest, wet meadow, marsh, seasonally flooded, floodplain forest, swamp and shallow water. Other classifications such as clouds and cloud shadows were not included in calculating landscape complexity (Table S3).

Field locations were determined using a global positioning system (Garmin Oregon 450t). Each field was assigned a unique code to designate a specific sample. The crops grown in the sampled and adjacent fields were also recorded. Adjacent field was the nearest field without crossing a hard barrier such as a road. The height of both the crops and vegetative edges were measured at 30 points along a transect between the pitfall traps using a measuring stick (Table S2).

### 2.2. Sampling Methods

From late May to mid-June of 2011 and 2012, invertebrates were sampled with sticky boards, sweep netting, and pitfall trapping, from each field once each year. Sticky boards and pitfall traps were positioned 28 May, 1 June, and 4 June 2011 and 26 May, 27 May, and 28 May of 2012, moving from south to north. Sticky boards and pitfall traps were placed at six locations per field, grouped equidistant from the ends of the field and adjacent to the FE; three in the FI and three on the FE spaced at 10 m intervals. Sampling sites in the FI were 10–15 m from the edge in the second equipment row (adjacent passage of the planter) and not in the turning row. Sites on the edge were 1–2 m from the FE within the vegetated edge. Sweep netting was conducted only in the FE to avoid damage to the crops.

Pitfall traps were 150 mL plastic cups with an aperture of 70 mm placed into the ground so that the mouths were flush with the ground and there was no discontinuity between the edge of the trap and the ground surface. Each trap was filled to ~2.5 cm with a solution of water and vinegar and a few drops of dish soap added to break the surface tension of the water. Ethylene glycol was not used because it attracted mammals to the traps during a pilot study. Pitfall traps were retrieved seven days after placement and contents placed in a labeled clear Ziploc bag containing 70% isopropyl alcohol and kept for future identification.

One sticky board (Sensor ~8 cm × 13 cm Yellow Monitoring Cards, GrowSmart, St. Louis, MO, USA), attached to a flag (~6 cm × 9 cm flag attached to a 76 cm long wire LimeGlo, Forestry Suppliers, Jackson, MS, USA) was placed adjacent to each pitfall trap. Boards were placed with a minimum of ½ the board above the vegetation. Boards were retrieved after two days, placed in a clear plastic cover and saved for future identification.

Sweep net sampling was conducted between the date when samples were placed and when pitfall samples were retrieved. A sweep net sample consisted of 30 strokes, 360° around the sweep netter in the FE near each of the pitfall traps for three samples total per field. The net was 38 cm in diameter with muslin netting (Forestry Suppliers). All sweep net samples were collected on sunny days between 10:00 a.m. and 2:00 p.m. with wind 0–3 as measured on the Beaufort scale. Invertebrates were placed in a “knockdown” jar containing chloroform soaked cotton for several minutes and then placed in a labeled clear plastic Ziploc bag containing 70% isopropyl alcohol and kept for future identification.

Invertebrates were examined using a binocular microscope. Ten percent of the samples were re-examined as quality control. An independent investigator adjudicated any conflicting identifications. Numbers of invertebrates smaller than 2 mm were estimated. Invertebrates larger than 2 mm were identified to lowest operational taxonomic unit (OTU) which in most cases was family, using taxonomic keys [31] and reference collections housed at the Illinois State Museum Research and Collections Center (ISM RCC). Some invertebrates were identified to order rather than family due to rarity, dominance of one family, or difficulty of identification.

### 2.3. Data Analysis

We used R (version 3.2.2, Available online: https://CRAN.R-project.org, accessed on 14 August 2015) and the package “lme4” [32,33]. We constructed six generalized linear mixed models (GLMM) assuming a Poisson error distribution for our global models. Response variables were either Taxonomic Richness (TR) or Diversity Index (DI). TR was the number of OTUs for each sample. DI was the exponentially transformed Shannon Wiener H' (e^H'^), making it Hill numbers of order 1 [34,35]. This transformation was done to ensure DI had the correct statistical characteristics for analysis [35]. Because of this transformation, DI can be interpreted as the number of abundant or common taxa within the sample [36]. We rounded DI to be able to apply a Poisson family GLMM. A sample is defined as one group of OTU’s per sampling method in each of three locations in the FI or FE per field per county.

The predictor variables that were handled as fixed factors in the construction of the models were measures of complexity at three different scales (500, 1000 and 6000 m) and the sample location within the field (either FE or FI). We tested the differences in the three counties at each of the three scales using ANOVA and *post-hoc* Tukey HSD. Since the complexity at 6000 m, 1000 m and 500 m was not independent, we constructed separate models for each scale. All other fixed variables were regarded as confounding variables and put in the models to correct for potential sources of bias due to the unequal spatial distribution of these variables. The confounding variables were checked with a correlation matrix and did not have high correlations. Fixed confounding variables were either quantitative or categorical. Quantitative variables (Table S2) included average vegetation height (cm), variation of vegetation height sd (cm), distance to the nearest non-arable space > 1 ha (m), width of the FE (m), length of the FE (m), and area of FI (ha). Categorical variables included crop in the FI (soybean or corn) and closest adjacent field (soybean, corn, grassland or developed). Developed included single homes, farm structures and parking areas. Since the 500 m, 1000 m and 6000 m buffers around the sample area that we used for measuring the complexity sometimes overlapped, introducing a potentially correlated effect of complexity on TR and DI between neighboring fields, we included the TR of the nearest field weighted by the area of overlap as a cofounding variable in the models. To study the difference in effect of confounding variable on FE and FI, we included the interaction between sample location (FE or FI) and all confounding variables of our models. There is a known relationship between the total number of individuals per sample, the sample size, and TR [37,38]. We applied a correction by including the log-transformed sample size, ln (abundance), in the models. We tested the importance of all predictor and confounding variables by applying a Likelihood Ratio Test (LRT) in which we removed each variable separately and tested the change in the likelihood of the models. The negative two times log likelihood ratio approaches a χ^2^-distribution [39].

Random factors were method of collection (sticky board, pitfall trap, and sweep net), and field within county within year of sampling. For testing the difference between counties, we removed county from the random variables and made it a fixed variable in the best fitting model of TR (the model at 1000 m scale). We then tested the impact of county with LRT. We followed the same procedure to determine the difference between years and sampling methods. For the purpose of fitting the models of TR, we added the identity of the sample (ID) as a random factor to the model. This makes the models for TR quasi-Poisson models [40]. Residuals were visually checked in all analyses for normality and equality of variance.

To find the simplest model for TR and DI, we first reduced the number of confounding variables per model stepwise based on a significant contribution of the variable to the model. We stopped this procedure when none of the remaining confounding variables could be taken out of the model without changing it significantly at the *p*-level of 0.10, so that even weakly confounding variables were still in the model. This left us with a relatively small model per level of scale for TR and DI, *i.e.*, with six separate parsimonious models. Then, using information theory [41,42], we selected the best fitting model for TR and DI. These two models will be presented in the results. Information on all six parsimonious models is presented in Supporting Information (Table S4a–f).

## 3. Results and Discussion

### 3.1. Results

#### 3.1.1. General Results

There were 890 samples collected by either pitfall trap, sticky board or sweep netting. This is less than the expected 900 because ten samples were lost due to animal disturbance. These ten samples were randomly distributed over the fields. A total of 155,460 invertebrates were identified to 138 different operational taxonomic units (OTUs) in the course of our study (Table S5). The three counties were significantly different in their landscape complexity (Table 1). The range of complexity varied between 5% and 79% non-agricultural area.

**Table 1:** Average ± standard error (se) of complexity for each of the three counties expressed as the percentage non-agricultural area of the area in a radius of 500, 1000, and 6000 m around the sampling location. Minimum and maximum percentages are between brackets. Results of the *F*-test on the difference between counties are given on the bottom line; * *p* < 0.05; *** *p* < 0.001. Results of Tukey HSD are given in Table S6.

County	500 m	1000 m	6000 m
Cass	48.3 ± 7.46 (5–78)	46.9 ± 7.47 (12–79)	33.8 ± 3.47 (19–49)
Christian	33.0 ± 3.70 (16–50)	27.9 ± 2.26 (15–37)	21.9 ± 1.54 (16–27)
Sangamon	39.7 ± 5.13 (17–63)	43.9 ± 1.34 (37–50)	36.0 ± 0.47 (32–37)
F _(2,27)_	1.85	4.99 *	11.80 ***

The stepwise reduction of the number of confounding variables per model resulted in different models for the three levels of scale of complexity (Table 2). Only field length and ln (abundance) remain present in all models. The interaction between complexity and location is significant in all models, but the main effects of location and complexity are only significant in the models for 1000 and 6000 m.

**Table 2:** Summary of the impact of the fixed effects on taxonomic richness (ln transformed) and diversity index at the different spatial scales. Pred. variables = predictor variables; Conf. variables = confounding variables. Confounding variables included crop in the field interior (FI; soybean or corn), closest adjacent field (soybean, corn, grassland or developed), area of FI (m^2^), width of the field edge (FE; m), length of the FE (m), distance to nearest non-arable (green) space > 1 ha, average vegetation height in both FI and FE (cm), variation of vegetation height (cm), correction factors for TR in the nearest sampling field weighted by buffer overlap and ln of abundance. For the predictor variables, * means that the estimated parameter is significantly different from zero. For the confounding variables it means that the variable could not be excluded from the model based on the LRT test, which means that either the main effect, the interaction effect with location or both effects are significant.

Fixed Effects	Taxonomic Richness			Diversity Index		
	500 m	1000 m	6000 m	500 m	1000 m	6000 m
**Pred. Variables**						
Complexity	-	*	*	-	*	*
Location (FE or FI)	-	*	*	-	*	*
Interaction	*	*	*	*	*	*
**Conf. Variables**						
Crop	-	*	*	*	*	-
Adjacent field	*	*	-	-	*	*
Field area	-	-	-	-	-	-
Field length	*	*	*	*	*	*
With of FE	-	-	-	-	-	-
Distance to green space	*	*	-	-	-	-
Height average	-	-	-	-	-	-
Height variability	-	-	-	-	-	-
TR nearest	-	-	-	-	-	-
Ln(abundance)	*	*	*	*	*	*

The three simplest models for TR were compared by applying information theory [41]. The TR model for the 1000 m and the 6000 m level of scale did not differ (Table 3). The same was done for DI and here the model for the 1000 m level of scale was clearly the best fitting (Table 4). The estimated fixed effects of all six models are given in Table S4a–f. We will use the 1000 m level models for further describing our results on TR and DI.

**Table 3:** Comparison of the three simplest models for taxonomic richness. Df: degrees of freedom of the model; AICc: corrected Akaike information criterion; Delta AICc: difference in AICc between the model and the model with the smallest AICc; AICcWt: model weight according to delta AICc; Cum. Wt: cumulative model weights; LL: Log Likelihood.

Complexity Scale	Df	AICc	Delta_AICc	AICcWt	Cum.Wt	LL
Model 1000	22	4749.85	0	0.56	0.56	−2352.34
Model 6000	14	4750.30	0.45	0.44	1	−2360.91
Model 500	18	4763.42	13.57	0	1	−2363.32

**Table 4:** Comparison of the three simplest models for diversity index. Df: degrees of freedom of the model; AICc: corrected Akaiki information criterion; Delta AICc: difference in AICc between the model and the model with the smallest AICc; AICcWt: model weight according to delta AICc; Cum. Wt: cumulative model weights; LL: Log Likelihood.

Complexity Scale	Df	AICc	Delta_AICc	AICcWt	Cum.Wt	LL
Model 1000	19	3545.72	0	0.98	0.98	−1753.42
Model 6000	15	3554.83	9.11	0.01	0.99	−1762.14
Model 500	13	3555.22	9.50	0.01	1	−1764.40

#### 3.1.2. Taxonomic Richness and Diversity

Taxonomic richness (TR) and Diversity (DI) was highest in Sangamon, followed by Cass and Christian counties (Table 5). TR and DI were higher in 2011 than 2012 (Table 6). Pitfalls sampled the most taxa, followed by sweep net samples (Table 7).

**Table 5:** Average ± se taxonomic richness (TR) and diversity index (DI) per sample in Sangamon (*n* = 298), Cass (*n* = 298), and Christian Counties (*n* = 294). LRT based on complete model (Table S7a,b); ** *p* < 0.01; *** *p* < 0.001.

County	TR	DI
Cass	9.87 ± 0.25	4.01 ± 0.17
Christian	9.30 ± 0.33	3.40 ± 0.12
Sangamon	11.86 ± 0.28	4.21 ± 0.15
LRT (Chi-sq, df = 3)	16.42 **	19.433 ***

**Table 6:** Average ± se taxonomic richness (TR) and diversity index (DI) per sample in 2011 and 2012. LRT based on complete model (Table S7c,d); *** *p* < 0.001.

Year	TR	DI
2011	12.27 ± 0.23	4.16 ± 0.13
2012	8.43 ± 0.23	3.59 ± 0.11
LRT(Chi-sq, df = 2)	81.00 ***	30.285 ***

**Table 7:** Average ± se, taxonomic richness (TR) and diversity index (DI) per sampling method. LRT based on complete model (Table S7e,f); *** *p* < 0.001.

Method	TR	DI
Pitfall	12.52 ± 0.26	5.06 ± 0.14
Sticky board	8.15 ± 0.23	2.21 ± 0.07
Sweeping net	10.47 ± 0.41	4.88 ± 0.20
LRT (Chi-sq, df = 3)	404.20 ***	347.29 ***

**Figure 2:**
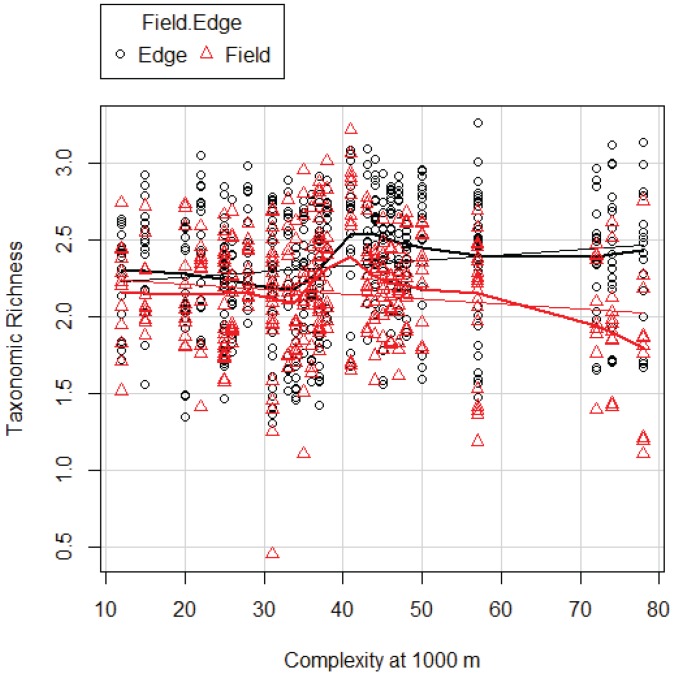
Taxonomic richness predicted by the best model for TR, Model 1000 (Table 8) in FE and FI as related to complexity at 1000 m. Thin line: linear regression line; thick line: non-linear regression line (LOESS curve). The y-axis is ln(TR).

**Figure 3:**
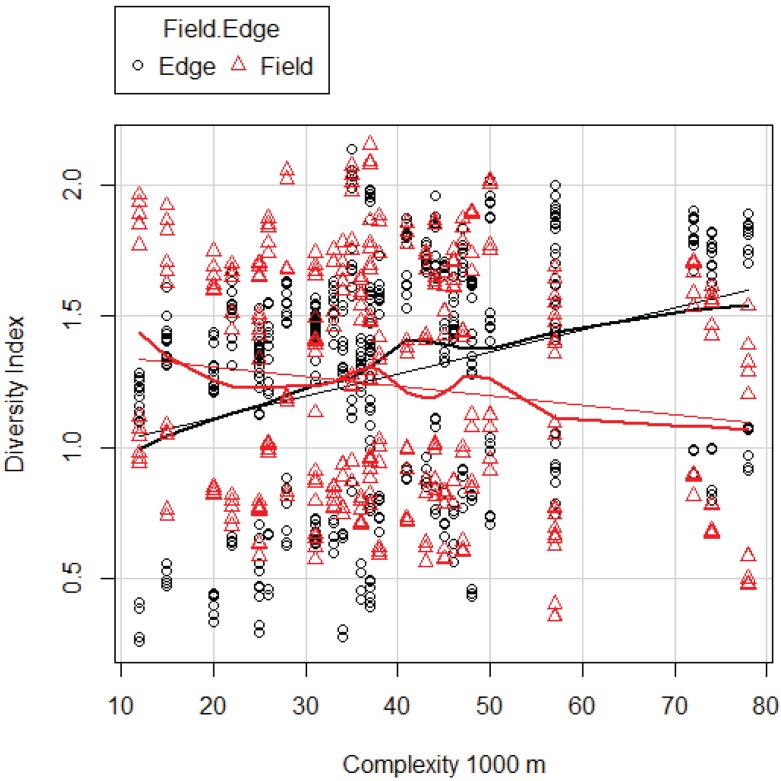
Diversity index (ln transformed) predicted by the best model for DI, Model 1000 (Table 9) in FE and FI as related to complexity at 1000 m. Thin line: linear regression line; thick line: non-linear regression line (LOESS curve).

TR was higher in the FE (overall average 11.08 ± 0.24) than in the FI (9.24 ± 0.24), and the model results show this difference is significant (Table S4b). In addition, TR is positively correlated with landscape complexity (Table S4b). But most striking is the strong interaction between location (either FE or FI) and complexity: TR decreases with increased landscape complexity in FI, while in contrast clearly it increases in FE (Figure 2, Table S4b). DI was higher in the FE (overall average 3.92 ± 0.11) than in the FI (3.80 ± 0.13). Although these differences appear small, they are significant (Table S4e). DI increases with complexity in FE and decreases in FI following the same pattern as TR (Figure 3, Table S4e).

**Table 8:** The best fitting model for TR (Model 1000, Table 3; model estimates in Table S5 b). Variables included Location (FE or FI), Complexity at 1000 m, crop in the FI (soybean or corn), closest adjacent field (soybean, corn, grassland or developed), length of the FE (m), distance to nearest non-arable (green) space > 1 ha, and sample size (ln abundance). The importance of the separate fixed factors were tested with a LRT. Df: degrees of freedom; AIC: Akaike information criterion, LL: Log Likelihood: Chi-sq: Chi-square (* *p* < 0.05; ** *p* < 0.01; *** *p* < 0.001).

TR	Df Model	AIC	LL	Chi-sq	Df chi	Probabilty
Complete model	22	4748.7	−2352.3			
Location (FE or FI)	13	4783.0	−2378.5	52.278	9	***
Complexity	20	4765.7	−2362.8	20.988	2	***
Crop	18	4751.5	−2357.8	10.857	4	*
Adjacent field	16	4752.4	−2360.2	15.718	6	*
Field length	20	4756.0	−2358.0	11.302	2	**
Distance to non-arable sp	20	4751.7	−2355.9	7.0544	2	*
Ln(abundance)	21	5132.2	−2545.1	385.52	1	***

**Table 9:** The best fitting model for DI (Model 1000, Table 4; model estimates in Table S4d–f). Variables included Location (FE or FI), Complexity at 1000 m, crop in the FI (soybean or corn), closest adjacent field (soybean, corn, grassland or developed), area of FI (ha), width of the FE (m), length of the FE (m), and sample size (ln abundance). Df: degrees of freedom; AIC: Akaike information criterion, LL: Log Likelihood: Chi-sq: Chi-square. The importance of the separate fixed factors were tested with a LRT (* *p* < 0.05; ** *p* < 0.01; *** *p* < 0.001).

DI	Df Model	AIC	LL	Chi-sq	Df chi	Probability
Complete model	22	3540.8	−1753.4			
Location (FE or FI)	12	3589.8	−1782.6	58.412	8	***
Complexity	18	3566.3	−1765.1	23.437	2	***
Crop	16	3547.9	−1757.9	9.0295	4	*
Adjacent field	14	3548.2	−1760.1	13.388	6	*
Field length	18	3550.5	−1757.2	7.65	2	**
Ln(abundance)	19	3556.6	−1759.3	11.72	1	***

As field length increased TR decreased in FI, but slightly increased in FE (Table S4b), while DI decreased both in FI and FE (Table S4e). TR and DI were influenced by the crops sown in the fields, with soybeans having the lowest TR and DI in FI and corn having the lowest TR and DI in FE (Table S4b,e). Crop in adjacent fields affected also TR and DI, with developed land having the lowest TR and DI in FI, but soybeans having the lowest TR in FE and corn having the lowest DI in FE (Table S4b,e). Soybeans in both the sampled FI and the adjacent field yielded the greatest TR: (Crop: 10.8 *vs*. 10.0; Adjacent Field: 11.3 *vs*. 10.5) and DI (Crop: 1.2 *vs.* 1.1; Adjacent Field: 1.2 *vs*. 1.1). Nearest non-arable space > 1 ha had a positive impact on the TR of FI and negative impact on FE but no impact on DI (Table S4b,e).

### 3.2. Discussion

Based on our analysis we developed a snapshot of invertebrate communities early in the growing season. We uncovered significant differences between fields and edges in the way these communities were affected by landscape complexity. We found it was affected by complexity at the landscape scale of 1000 m and 6000 m. DI was affected by complexity at 1000 m. Both TR and DI increased with increasing complexity in FE and surprisingly decreased with increasing complexity in FI. Here we showed that the patterns of both TR and DI in fields and edges were identical as far as the relationship with landscape complexity was concerned.

We proposed several hypotheses that guided our study design and data collection:

- TR and DI would be greater in the edges than the fields.
- TR and DI would be greater as landscape complexity is greater, *i.e.*, the relative non-agricultural area is greater. This degree of increase could be different for TR and DI.
- Local factors, which we addressed as confounding variables, may have a significant effect on TR and DI independent of landscape complexity.

#### 3.2.1. Difference between FE and FI

The difference in TR between edges (FE) and fields (FI) seems small, however was statistically significant. Greater differences have been reported in a south African study in corn fields [43] and in European studies that examined a large variety of field edges as they relate to organic farming [44], ecological compensation meadows [45] and ditch banks [20].

Boundaries between “habitat” and “non-habitat” are often clearly identifiable to the human eye and presumably also to invertebrates [46]. The landscapes in our study had a large percentage of areas that would be considered usable habitat (forests, grassland, *etc.*) for invertebrates with the agricultural FI presumably at the lower end of any habitat ranking (Table 1). It is therefore not surprising that FE had more invertebrates than FI. Agricultural FI recently disturbed by planting would appear to be even more lacking in habitat value. Our results, however, are consistent with the positive values of soil loosened by planting, warmed by exposure to sunlight, and drained of heavy spring rains. Many invertebrates deposit eggs into the soil; larvae feed on underground roots and detritus; they pupate and emerge as adults to mate. Ants and the larvae of some beetles, moths, flies and worms transport below ground materials to above ground consumers [47]. The agricultural FI thus have habitat value and are not “non-habitat”. Invertebrates that occupy the FI are obviously “adapted” to high disturbance levels and monoculture vegetation. They are frequently generalists that have a small body size and short life-cycle [48]. These generalists are successful and likely account for TR in the FI. Species requiring pristine environments, undisturbed habitats or that have limited dispersal ranges can be expected to be rare in the FI, but could find conditions in the FE that provide a great range of possible habitable environments with varied vegetation type, height, edge width, *etc.*

TR and DI are highly correlated but give different information. TR gives equal weight to rare taxa as common taxa; DI gives more emphasis on common taxa with little contribution from rare taxa. DI is a relative measure of evenness [36]. This is an important component of biodiversity. Having great numbers of a single pest species and few predator species may result in the same TR as having balanced numbers of predator and prey. Knowing that the same patterns persist with TR and DI for FE and FI allows us to address important ecological food webs without harming agricultural yields.

Vegetation in the FE and non-arable areas was not classified as native or non-native in our study. Presumably the non-arable areas had mostly native species, while the FE had varying amounts of native plants. Native invertebrates have evolved in step with the native plants and would optimize interactions. The crops in the FI are non-native. Providing a complex native habitat should provide opportunities for native invertebrate species. Future studies should assess the relationship of invertebrate diversity to native vegetation in the FE.

Differences in agricultural practices may account for divergence from European studies. Studies of FI and FE in Europe deal with a vastly different agricultural system than we studied in central Illinois. Researchers of European agricultural systems have studied cereal crops, peas, potatoes, and sugar beets with small scale crop rotation [22,49]. GMO crops are infrequently sown and are prohibited in some nations [50] while they are the norm for Illinois [51]. Some FI are quite large in our study area (e.g., 117 ha) while many European study field interiors are considerably smaller, e.g., 22–30 ha (e.g., [52]). Field edges in Europe are relatively stable and consistent temporally [20]. In our study area, FE are frequently mown, treated with herbicide, exposed to de-icing chemicals and burned, and therefore show little contrast from FI in our study or in field edges in Europe.

Our study was conducted during a two-week period in early summer, not long after crops had been sown. The period before our study included major disturbance of the soil from cultivation in the FI and potential movement of invertebrates between FI and FE. Invertebrates vary in their timing of emergence from diapause. The crops were growing rapidly but not yet shading the ground between plants as occurs later in the growing season. Patterns observed at this time may not be the same as patterns later in the growing season. Future studies should look at TR and DI across the growing season.

Agricultural areas in many parts of Europe have been active for many centuries if not millennia and in Illinois somewhat less than two centuries [53,54]. Data from European studies may not be transferable to studies in Illinois and vice versa. That does not mean, however, that the management techniques that are shown to be effective in Europe would not be equally effective in Illinois. Practices that might be beneficial in Illinois include “beetle banks” [14], reduction of chemical applications in conservation headlands and field margins [55], and Ecological Conservation Area wildflower strips [56].

#### 3.2.2. Landscape Complexity

Most of the fields of our study (*n* = 30) were located in an area (6000 m scale) of high non-agricultural complexity of 16%–30% (*n* = 14), 30%–40% (*n* = 13) and >40% (*n* = 3). This is unexpected in an area known as the “corn desert” [57] and may be related to our selection of structurally diverse FE in proximity to natural areas. Assuming that the intermediate landscape complexity hypothesis [3] is also applicable in the agricultural areas of the Midwest, this would mean that agri-environmental measures to increase biodiversity would have little effect in our study area: the TR and DI are already optimal for an agricultural area. This idea is supported by the fact that the TR we found in the FI is relatively high and close to the TR in the FE (*ca*. 9 *vs.* *ca*. 11 taxa per sample on average). However, it should be noted that our study areas may not be representative of the rest of the Midwest.

Local communities are firstly dependent on the regional pool of species [58]. Local TR is expected to be larger within areas of greater landscape complexity [22], because increased complexity increases the regional pool from which to draw local communities [28]. Therefore the higher TR that we found in the FE in more complex landscapes fits this species pool hypothesis.

However, according to the species pool hypothesis, we would also expect higher TR in the FI. The pattern that emerges in our data shows that as TR in the FE increases, the TR in the FI decreases. There are several possible explanations of this deviation from expectation, which could be examined in further studies. First, because of the higher TR in the more complex landscapes, the predation pressure on invertebrates could be higher, either by other invertebrates or by vertebrates that were not measured by us [54,59]. In the FE more invertebrate species would be able to escape this increased pressure because of the higher vegetation density, while in the fields the invertebrates are more vulnerable to predation. This would fit Tscharntke and coworkers’ [60] hypothesis that landscape complexity provides spatial and temporal insurance, which would mean in this case a more efficient regulation of pest species populations in the FI.

Second, another explanation is that invertebrates prefer the FE habitat and non-agricultural landscape elements even when they are able to occupy the FI niche. In this case, the data may reflect species moving into the FI only when they have no other option, e.g., when individual numbers are high in the FE or resources are depleted, but no escape to other landscape elements than FI is possible. When there is other, more suitable habitat available, that is where invertebrates will occur.

Third, it is intriguing to consider that plant-to-plant interactions in more complex areas may provide a defense for the FI [61,62]. This might involve a signal sent by plants in the edge in response to herbivory being received by the crops in the FI [63]. Because the FI are a monoculture, the response spreads through the entire FI providing protection against the herbivores either repelling the herbivores or calling predators or perhaps both [64,65]. This effect could be masked in the FE by the dense vegetation.

We tested whether the study areas within the three counties varied significantly in their complexity (Table 1). The study areas were not randomly selected; they were selected because of their proximity to non-arable land within the agricultural landscape and did not include urban areas within the buffer circles. The three counties were typical of the Illinois landscape, but the study areas selected were probably more complex than the remainder of the land in the counties. These areas were not necessarily representative of either the rest of county, state or even Midwest.

#### 3.2.3. Confounding Variables

A drought period began in the summer of 2011 and continued through 2012 with higher than normal temperatures and lower than normal rainfall. We tested if the difference in TR and DI was significantly different between years (Table 6). We do not know if the highly significant difference between years was typical of the normal variability of invertebrate populations or a product of the drought conditions.

We measured a number of confounding variables that we felt might impact TR and DI. Crops in the study areas were GM soybean and corn. Corn is usually the first crop to be sown when the fields dry out and start to warm. Soybeans are planted later. The planting dates and subsequent plant growth and shading may have influenced colonization by invertebrates. Price [66] found that herbivores colonized the soybeans first with no appreciable increase in parasites and predators until the canopy had developed. Botha [43] found that biodiversity loss was apparent if corn fields were within 30 m of the field margins being sampled. Therefore it was no surprise that crop had an effect in our study.

All FI were tilled before planting and may or may not have been recently treated with glyphosate (broad spectrum herbicide). Herbicides vary in their impact on invertebrates and the impact often depends on the timing and context of the application [67,68]. Invertebrates vary greatly in their mobility and dispersal ability. In agricultural landscapes with high disturbance particularly in the planting and harvesting phases the dispersal technique is crucial for survival [3]. In addition, crossing hard barriers, such as roadways, limits the mobility of arthropods [69]. These issues are outside the scope of this study but should be acknowledged as having an impact.

As the length of the FE increased, the TR and DI decreased. The edge along the fields may serve as a corridor for migration as well as a refuge during episodes of disturbance. The distance to additional field edges increases vulnerability of invertebrates with low mobility.

The distance to the nearest non-arable space > 1 ha was important to TR but had no significant impact on DI. We did not collect from the nearest non-arable space and cannot say how the TR and DI compared to our study fields. Gonzalez [27] found both forest cover and proximity affected arthropod assemblages in soybean fields in central Argentina.

We measured a number of other factors which did not significantly contribute to our findings (Table 2). These included the size of the agricultural field, the width of the FE, the average vegetation height, vegetation standard deviation (sd) and soil type. These were local factors within the agricultural landscape that affect other groups of organisms such as birds [70,71], and mammals [72,73], but not some reptiles [74].

## 4. Conclusions

Our data indicate that invertebrate diversity responds to characteristics operating at both field and landscape levels. For FI and FE, habitat quality (in our analyses shown as the significance of confounding variables) had multiple effects. Research that considers the agricultural landscape strictly as a mosaic of habitat and non-habitat fails to recognize the utilization and possible enhancement of biodiversity provided by the managed FE since these may have considerable TR and DI of invertebrates. Knowing that landscape complexity is relatively high in some areas of central Illinois as compared to European landscapes, additional investigation is needed to determine whether there are special opportunities to enhance biodiversity in the agricultural landscape of central Illinois. Agri-environment schemes of the European Union have sometimes been shown to be effective in improving biodiversity [75,76]. Keeping the large scale complexity of the landscape that currently exists is clearly an important conservation strategy to preserve invertebrate populations. There seems to be no detrimental effects from an agriculture point of view, because a more complex landscape does not result in higher TR in FI. Planting FE with native plants is an easy step in providing habitat for native insects that could be tested.

## Supplementary Files

Supplementary File 1
