# Supplementary material for: The Impact of Landscape Complexity on Invertebrate Diversity in Edges and Fields in an Agricultural Area
Source: Insects. 2016 Feb 3;7(1):7. doi: 10.3390/insects7010007 (PMC4808787; doi:10.3390/insects7010007)
Supplement: Supplementary File 1 [file insects-07-00007-s001.pdf]

Supplementary Materials: The Impact of Landscape Complexity on Invertebrate Diversity in Edges and Fields in an Agricultural Area

Tracy R. Evans, Meredith J. Mahoney, Everett D. Cashatt, Jinze Noordijk, Geert de Snoo and C.J.M. Musters

1. Experimental Section

1.1. Study Area

We selected ten agricultural fields mostly seeded in a 2–3 years corn (*Zea mays*) and soybean (*Glycine max*) planting rotation in each of three counties for a total of 30 fields (Supporting Information, Table S1). Fields were visually selected for varied edge structure. The average field size was 28 ha with a range from 1–117 ha (Table S2). Fields differed in their surrounding structural complexity, ranging from simple landscapes with a relatively high percentage of arable land, to complex landscapes with a relatively low percentage of arable land and a large proportion of semi-natural land cover and other land use types (Table S3). The edge structure and vegetation ranged from closely mown grass monoculture to shrubby vegetation more than a meter in height (Table S2). Permission to access the fields was obtained from land managers and landowners (in many cases the landowner was different from the land manager). Vegetation in the FE was managed by various entities including the landowner, land manager, and township employees and consisted of a variety of mowing and herbicide regimes. Prior to the start of the study, FIs had been seeded with genetically modified (Roundup Ready) corn or soybeans by the landowners or managers (Table S1). Roundup Ready seeds are modified to be resistant to glyphosate type herbicides that are used to control weeds.

Table S1. Field characteristics of Sangamon County sites (SANG), Cass County sites (CASS) and Christian County sites (CHRIS) Field size was measured in ha and field length and the width of the field edge (FE) measured in m. Soil type was determined using Natural Resource Conservation Service soil maps. Latitude and longitude were determined using a Garmin Oregon 450t Global Positioning System (GPS) unit.

Site	Field	Crop 2011	Adjacent Field	Crop 2012	Adjacent Field	Field Size	Field Length	FE Width	Soil type	Latitude	Longitude
SANG	1	soybeans	grassland	grassland	grassland	13.3	811	5	Proctor silt loam	39°45'52 33" N	89°26'40 07" W
SANG	2	soybeans	soybeans	corn	grassland	117	463	22	Proctor silt loam	39°45'49 62" N	89°26'42 90" W
SANG	3	soybeans	soybeans	corn	corn	9.5	225	3	Proctor silt loam	39°45'51 63" N	89°26'57 68" W
SANG	4	soybeans	soybeans	corn	corn	117	281	15	Vesser silt loam	39°45'45 96" N	89°27'8 56" W
SANG	5	soybeans	soybeans	corn	corn	9.5	227	10	Kendall silt loam	39°45'56.63" N	89°27'12.58" W
SANG	6	corn	corn	soybeans	corn	18	230	11	Plano silt loam	39°45'31.65" N	89°26'25.70" W
SANG	7	corn	corn	soybeans	soybeans	4.1	451	4	Kendall silt loam	39°45'33.45" N	89°26'29.45"W
SANG	8	soybeans	soybeans	corn	grassland	1	150	33	Sand	39°45'26.45" N	89°26'40.88" W
SANG	9	corn	developed	soybeans	developed	4.3	172	16	Plano silt loam	39°44'53.93" N	89°26'40.84" W
SANG	10	soybeans	corn	corn	corn	26.7	392	4	Alvin fine sandy loam	39°45'25.29" N	89°26'35.91" W
CASS	1	corn	soybeans	corn	soybeans	49	1203	3	Ipava silt loam	39°56'43.81" N	90°3'22.38" W
CASS	2	soybeans	corn	soybeans	corn	5	151	42	Rozetta silt loam	39°56'44.65" N	90°3'56.20"W
CASS	3	corn	grassland	corn	grassland	25	404	3	Ipava silt loam	39°56'59.54" N	90°3'54.95" W
CASS	4	corn	corn	corn	corn	10.2	310	58	Rozetta silt loam	39°58'6.94" N	90°3'21.74"W
CASS	5	corn	corn	corn	corn	8.2	500	21	Ipava silt loam	39°58'31.91" N	90°3'21.21" W
CASS	6	corn	corn	corn	corn	11.8	387	11	Rozetta silt loam	39°58'56.08" N	90°3'20.98" W
CASS	7	soybeans	corn	soybeans	corn	3.1	182	37	Ipava silt loam	39°58'57.12" N	90°3'18.59" W
CASS	8	soybeans	grassland	soybeans	grassland	3.2	319	33	Fayette silt loam	39°59'57.77" N	90°3'55.40" W
CASS	9	soybeans	grassland	soybeans	grassland	10.6	264	34	Fayette silt loam	40°00'50.00" N	90°3'58.24" W
CASS	10	corn	grassland	corn	grassland	2.3	215	16	Rozetta silt loam	40°00'21.21" N	90°3'56.83" W
CHRIS	1	corn	grassland	corn	corn	105.6	533	9	Ipava silt loam	39°39'20.88" N	89°30'57.79" W
CHRIS	2	corn	grassland	soybeans	corn	20.2	585	3	Ipava silt loam	39°39'18.92" N	89°31'8.64" W
CHRIS	3	soybeans	soybeans	soybeans	corn	19.6	298	9	Buckhart silt loam	39°39'20.32" N	89°29'56.54" W
CHRIS	4	corn	soybeans	soybeans	soybeans	31.3	777	3	Ipava silt loam	39°39'17.76" N	89°29'41.37" W
CHRIS	5	soybeans	grassland	soybeans	grassland	31	773	3	Keomah silt loam	39°38'52.66" N	89°27'51.46" W
CHRIS	6	corn	grassland	corn	grassland	105.6	811	3	Rozetta silt loam	39°39'42.58" N	89°24'58.88" W
CHRIS	7	corn	developed	corn	developed	20.2	463	3	Rozetta silt loam	39°39'43.96" N	89°24'43.97" W
CHRIS	8	corn	grassland	corn	grassland	19.6	225	37	Rozetta silt loam	39°39'44.01" N	89°24'23.48" W
CHRIS	9	corn	grassland	corn	grassland	31.3	281	6	Rozetta silt loam	39°39'44.03" N	89°24'11.20" W
CHRIS	10	corn	grassland	corn	corn	31	227	23	Elco silt loam	39°39'38.86" N	89°24'16.85" W

Table S2. Average ± standard error (se) of fixed quantitative variables (FE; field edge, FI: field interior). Minimum and maximum are between brackets.

Field Characteristics	$\bar{x}$ ± se (Range)
Average FE vegetation height (cm)	70.6 ± 5.96 (10–130)
Average FI vegetation height (cm)	27.0 ± 3.90 (5–80)
Vegetation height FE (cm)	19.6 ± 5.36 (0–152)
Vegetation height FI (cm)	0.1 ± 0.12 (0–3.6)
Distance to nearest non-arable space >1 ha (m)	115.4 ± 19.55 (10–450)
Width of the FE (m)	16.0 ± 2.71 (3–58)
Length of FE (m)	410.3 ± 45.66 (150–1203)
Field area (ha)	28.8 ± 6.36 (1–117)
Complexity 6000 (%)	30.6 ± 1.67 (16–49)
Complexity 1000 (%)	39.6 ± 2.98 (12–78)
Complexity 500 (%)	40.3 ± 3.35 (5–78)

Table S3. Land Cover Categories used to determine complexity.

**Agricultural Land**
Corn
Soybeans
Winter Wheat
Other Small Grains and Grassland
Winter Wheat/Soybeans
Other Agriculture
Rural Grassland
**Forested Land**
Upland
Partial Canopy/Savannah Upland
Coniferous
**Urban Land**
High Density
Low/Medium Density
Urban Open Space
**Wetland**
Shallow Marsh/Wet Meadow
Deep Marsh
Seasonally/Temporarily Flooded
Floodplain Forest
Swamp
Shallow Water
**Other**
Surface Water
Barren and Exposed Land
Clouds
Cloud Shadows

Table S4. Models and summary tables for taxonomic richness (TR) and diversity index (DI). Variables included Location (FI or FE); the proportion of non-agricultural area at three different scales (6000 m, 1000 m, and 500 m), Crop in the FI (Soybeans, corn, grassland), closest Adjacent Field (Soybeans, corn, grassland or developed), length of the FE (m), distance to nearest non-arable space > 1 ha (Green1ha), and a correction factor for sample size (ln Abundance). Our random factors were method of collection (sticky board, pitfall trap, or sweep net) and field within county within year (CountyYearField). We included the identity of the sample (fID) to fit a quasi-Poisson distribution for TR. Signif. codes: $p <$ 0.001: '***'; $p >$ 0.001: '**'; $p >$ 0.01: '*'; $p >$ 0.05: '.' $p >$ 0.1: NS.

a. TR ~ Location * (Complexity6000 + Crop + Length) + ln Abundance + (1|CountyYearField) + (1|Method) + (1|fID).

Variables	Estimate	Std. Error	*z* value	Pr(>|z|)	
Intercept (FE)	0.9818	0.2816	3.487	0.0005	***
Location FI	0.5486	0.2339	2.345	0.0190	*
Complexity6000	0.0068	0.0028	2.442	0.0146	*
Crop Corn	−0.0630	0.1890	−0.333	0.7388	NS
Crop Soybeans	−0.0613	0.1892	−0.324	0.7460	NS
Length	−0.0002	0.0001	−2.204	0.0275	*
Ln Abundance	0.2792	0.0132	21.225	$<2^{-16}$	***
Location FI:Complexity6000	−0.0131	0.0029	−4.514	6.36^{-06}	***
Location FI: Crop Corn	−0.3412	0.1856	−1.839	0.0660	.
Location FI: Crop Soybeans	−0.2092	0.1857	−1.126	0.2600	NS
Location FI: Length	0.0002	0.0001	2.046	0.0408	*

b. TR ~ Location * (Complexity1000 + Crop + Adjacent Field + Length + Green1ha > 1ha) + ln Abundance + (1|CountyYearField) + (1|Method) + (1|fID).

Variables	Estimate	Std. Error	*z* value	Pr(>|z|)	
Intercept (FE)	1.0402	0.2794	3.723	0.0002	***
Location FI	0.5383	0.2364	2.277	0.0228	*
Complexity1000	0.0044	0.0017	2.578	0.0099	***
Crop Corn	−0.1396	0.1883	−0.742	0.4583	NS
Crop Soybeans	−0.1893	0.1912	−0.99	0.3222	NS
Length	−0.0003	0.0001	−2.593	0.0095	**
Adjacent Field Hay	0.0457	0.0869	0.526	0.5986	NS
Adjacent Field Corn	0.0524	0.0843	0.622	0.5341	NS
Adjacent Field Soybeans	0.2016	0.0998	2.02	0.0434	*
Green1ha	0.0002	0.0003	0.745	0.4560	NS
ln Abundance	0.2834	0.0133	21.381	$<2^{-16}$	***
Location FI: Complexity1000	−0.0083	0.0019	−4.487	0.00001	***
Location FI: Crop Corn	−0.1768	0.1918	−0.922	0.3568	NS
Location FI: Crop Soybeans	−0.0104	0.1944	−0.053	0.9575	NS
Location FI: Length	0.0004	0.0001	3.051	0.0023	**
Location FI: Adjacent Field Hay	−0.2059	0.0914	−2.252	0.0243	*
Location FI: Adjacent Field Corn	−0.2770	0.0886	−3.128	0.0018	**
Location FI: Adjacent Field Soybeans	−0.2784	0.1047	−2.66	0.0078	**
Location FI: Green1ha	−0.0008	0.0003	−2.661	0.0078	**

c. TR ~ Location * (Complexity500 + Adjacent Field + Length + Green1ha) + ln Abundance + (1|CountyYearField) + (1|Method) + (1|fID).

**Variables**	**Estimate**	**Std. Error**	***z* value**	**$\Pr(>\lvert z\rvert)$**	
Intercept (FE)	1.03^{-00}	1.97^{-01}	5.222	1.77^{-07}	***
Location FI	1.45^{-01}	1.12^{-01}	1.289	0.1972	NS
Complexity500	1.65^{-03}	1.46^{-03}	1.126	0.2600	NS
Length	-3.52^{-04}	1.19^{-04}	−2.962	0.0031	**
Green1ha	7.83^{-05}	2.90^{-04}	0.27	0.7870	NS
Adjacent Field Hay	6.87^{-02}	8.77^{-02}	0.783	0.4336	NS
Adjacent Field Corn	4.38^{-02}	8.55^{-02}	0.512	0.6084	NS
Adjacent Field Soybeans	1.88^{-01}	9.61^{-02}	1.958	0.0502	.
ln Abundance	2.80^{-01}	1.34^{-02}	20.945	$<2^{-16}$	***
Location FI: Complexity500	-2.83^{-03}	1.54^{-03}	−1.845	0.0650	.
Location FI: Length	5.17^{-04}	1.24^{-04}	4.171	3.03^{-05}	***
Location FI: Green1ha	-7.66^{-04}	2.99^{-04}	−2.566	0.0103	*
Location FI: Adjacent Field Hay	-2.22^{-01}	9.13^{-02}	−2.427	0.0152	*
Location FI: Adjacent Field Corn	-2.45^{-01}	8.87^{-02}	−2.756	0.0059	**
Location FI: Adjacent Field Soybeans	-2.04^{-01}	9.95^{-02}	−2.052	0.0402	*

d. DI ~ Location * (Complexity6000 + Adjacent Field + Field Length) + ln Abundance + (1|CountyYearField) + (1|Method).

**Variables**	**Estimate**	**Std. Error**	***z* value**	**$\Pr(>\lvert z\rvert)$**	
Intercept (FE)	1.3829	0.2717	5.09	3.57^{-07}	***
Location FI	0.3632	0.1771	2.051	0.0403	*
Complexity6000	0.0105	0.0037	2.875	0.0040	**
Adjacent Field Hay	0.0353	0.1241	0.284	0.7761	NS
Adjacent Field Corn	0.1032	0.1201	0.859	0.3902	NS
Adjacent Field Soybeans	0.0881	0.1359	0.649	0.5167	NS
Length	−0.0004	0.0001	−3.074	0.0021	**
ln Abundance	−0.0699	0.0193	−3.625	0.0003	***
Location FI: Complexity6000	−0.0114	0.0043	−2.654	0.0080	**
Location FI: Adjacent Field Hay	−0.1154	0.1407	−0.82	0.4124	NS
Location FI: Adjacent Field Corn	−0.2239	0.1361	−1.644	0.1001	NS
Location FI: Adjacent Field Soybeans	0.0476	0.1525	0.312	0.7547	NS
Location FI:Length	0.0004	0.0002	2.724	0.0065	**

e. DI ~ Location * (Complexity1000 + Crop + Adjacent Field + Field Length) + ln Abundance + (1|CountyYearField) + (1|Method).

**Variables**	**Estimate**	**Std. Error**	***z* value**	**$\Pr(>\lvert z\rvert)$**	
Intercept (FE)	1.5701	0.3802	4.129	3.64^{-05}	***
Location FI	0.7754	0.3448	2.249	0.0245	*
Complexity1000	0.0066	0.0023	2.929	0.0034	**
Crop Corn	−0.2169	0.2413	−0.899	0.3689	NS
Crop Soybeans	−0.2407	0.2460	−0.978	0.3279	NS
Adjacent Field Hay	0.0666	0.1206	0.552	0.5809	NS
Adjacent Field Corn	0.1962	0.1169	1.679	0.0931	.
Adjacent Field Soybeans	0.2158	0.1392	1.551	0.1210	NS
Length	−0.0004	0.0002	−2.749	0.0060	**
ln Abundance	−0.0662	0.0194	−3.42	0.0006	***
Location FI:Complexity1000	−0.0131	0.0028	−4.748	2.06^{-06}	***
Location FI: Crop Corn	−0.1715	0.2654	−0.646	0.5180	NS
Location FI: Crop Soybeans	0.0419	0.2709	0.155	0.8779	NS
Location FI: Adjacent Field Hay	−0.1505	0.1374	−1.095	0.2737	NS
Location FI: Adjacent Field Corn	−0.3752	0.1340	−2.801	0.0051	**
Location FI: Adjacent Field Soybeans	−0.2045	0.1573	−1.3	0.1936	NS
Location FI: Length	0.0003	0.0002	1.849	0.0645	.

f. DI ~ Location * (Complexity500 + Crop + Field Length)+ ln Abundance + (1|CountyYearField) + (1|Method).

Variables	Estimate	Std. Error	*z* value	Pr(>|z|)	
Intercept (FE)	2.0029	0.3698	5.416	6.10^{-08}	***
Location FI	0.2479	0.3263	0.76	0.4473	NS
Complexity500	0.0013	0.0018	0.692	0.4889	NS
Crop Corn	−0.2543	0.2461	−1.033	0.3014	NS
Crop Soybeans	−0.1969	0.2473	−0.796	0.4260	NS
Length	−0.0006	0.0001	−3.983	6.79^{-05}	***
ln Abundance	−0.0712	0.0192	−3.714	0.0002	***
Location FI: Complexity500	−0.0071	0.0021	−3.302	0.0010	***
Location FI: Crop Corn	−0.1781	0.2690	−0.662	0.5080	NS
Location FI: Crop Soybeans	−0.0422	0.2702	−0.156	0.8757	NS
Location FI: Length	0.0006	0.0002	3.584	0.0003	***

Table S5. Presence/Absence of taxa and TR for the three counties for study years 2011 and 2012. Locations are Sangamon County (SANG), Cass County (CASS) and Christian County (CHRIS). * indicates presence.

Taxa	2011			2012		
	SANG	CASS	CHRIS	SANG	CASS	CHRIS
Class Oligochaeta: Earthworms	*	*		*		
Class Gastropoda: Snails	*	*		*		
Order Araneae: Spiders						
Araneidae: Orb-weavers	*			*	*	
Clubionidae: Sac Spider			*			
Gnaphosidae: Parson Spider	*	*	*	*	*	*
Linyphiidae: Sheet Web Spiders	*	*	*	*	*	*
Lycosidae: Wolf Spiders	*	*	*	*	*	*
Pisauridae: Nursery Web Spiders	*	*		*	*	*
Salticidae: Jumping Spiders	*	*	*	*	*	*
Tetragnathidae: Long-jawed Spiders	*			*		
Thomisidae: Crab Spiders	*	*	*	*	*	*
Order Opiliones: Harvestmen	*	*	*	*	*	*
Order Acari: Ticks	*	*	*	*	*	
Order Isopoda: Isopods						
Common Pillbug	*	*	*	*	*	*
Order Diplopoda: Millipedes	*	*	*	*	*	*
Order Chilopoda: Centipedes	*	*		*	*	*
Order Collembola: Springtails	*	*	*	*	*	*
Order Odonata						
Dragonflies	*	*	*	*	*	
Damselflies		*			*	
Order Orthoptera						
Crickets	*	*	*	*	*	*
Grasshoppers	*	*	*	*	*	*
Order Phasmatodea: Walkingsticks						
Heteronemiidae: Stick Bug				*		
Order Plecoptera: Stoneflies						
Leuctridae	*					
Perlidae			*			
Order Mantidea: Mantids		*			*	
Order Blattaria: Cockroaches						
Blattidae: Cockroaches	*				*	
Order Hemiptera:True Bugs						
Alydidae: Broad-headed Bugs					*	
Anthocoridae: Minute Pirate Bugs	*		*	*	*	*
Aphidae: Aphids	*	*	*	*	*	*
Cercopidae: Spittlebugs	*	*	*			
Cicadellidae: Leafhoppers	*	*	*	*	*	*
Cicadidae: Cicadas	*					

Coreidae: Squash Bugs			*			
Cydnidae : Burrower Bugs	*					
Lygaeidae: Seed Bugs:	*		*	*	*	*
Membracidae: Treehoppers	*	*	*			
Miridae: Plant Bugs	*	*	*	*	*	*
Nabidae: Damsel Bugs	*	*	*			
Pentatomidae: Stink Bugs				*		
Reduviidae : Assassin Bugs	*	*	*	*	*	*
Rhyparochromidae: Dirt-coloredSeed Bug			*			
Scutellaridae: Shield-backed Bugs	*					
Thyreocoridae: Negro Bugs	*	*	*	*	*	*
Tingidae: Lace Bugs	*		*			
Order Thysanoptera: Thrips	*	*	*	*	*	*
Order Coleoptera: Beetles						
Anthicidae: Ant-like flower Beetles	*					
Buprestidae: Jewel Beetles		*			*	
Cantharidae: Soldier Beetles	*	*	*	*		*
Carabidea: Ground Beetles	*	*	*	*	*	*
Cerambycidae: Long-horned Beetles	*					
Chrysomelidae: Leaf Beetles	*	*	*	*	*	*
Cicindelidae: Tiger Beetles	*	*				
Cleridae: Checkered Beetles				*	*	*
Coccinellidae: Lady Beetles	*	*	*	*	*	*
Curculionidae: Weevils	*	*	*	*	*	*
Elateridae: Click Beetles	*	*	*	*	*	*
Erotylidae: Pleasing fungus beetles	*	*	*	*	*	*
Histeridae: Hister Beetles	*	*	*	*	*	
Lampyridae: Fireflies	*	*	*	*		
Meloidae: Blister Beetles		*	*	*	*	
Mordellidae: Tumbling Flower Beetles:	*	*	*	*	*	*
Nitidulidae: Sap Beetles	*		*		*	
Pyrochroidae: Fire-colored beetles		*				
Scaphidiidae: Shining fungus beetles		*	*	*	*	*
Scarabaeidae: Scarab Beetles	*	*	*	*	*	*
Silphidae: Carrion Beetles	*	*	*	*	*	*
Staphylinidae: Rove Beetles	*	*	*	*	*	*
Tenebrionidae: Darkling Beetles	*		*	*		*
Trogidae: Trox Beetles	*			*	*	*
Order Neuroptera: Antlions, Lacewings	*	*	*	*		
Hymenoptera:Wasps, Bees, Ants						
Andrenidae:Mining Bees	*			*	*	*
Apidae: Honey Bees	*	*	*	*	*	*
Braconidae: Parasitic Wasps	*	*	*	*	*	*
Chalcidae: Parasitic Wasps		*		*		*
Chrysididae : Cuckoo Wasps				*	*	*
Colletidae: Plasterer and Yellow- faced Bees			*			
Formicidae: Ants	*	*	*	*	*	*
Halictidae: Sweat Bees	*	*	*	*	*	*
Ichneumonidae: Ichneumon Wasps	*	*	*	*	*	*
Megachilidae: Leafcutter Bees	*	*	*	*	*	*
Mellittidae: Oil-Collecting Bees	*					
Mutillidae: Velvet Ants				*		
Pompilidae: Spider Wasps				*		
Siricidae: Horntails	*			*		
Sphecidae: Thread-Waisted Wasps	*			*	*	
Vespidae: Yellowjackets, Hornets, Paper Wasps; Potter, Mason and Pollen Wasps	*	*	*			*
Lepidopetera: Butterflies and Moths						
Arctiidae: Tiger Moths		*				
Hesperiidae: Skippers	*	*	*	*		*
Lycaenidae: Copper/Gossamers	*	*	*	*	*	*
• Micro-lepidoptera	*	*	*	*	*	*
Noctuidae: Owlet Moths	*	*	*	*	*	
Nymphalidae: Brush-footedButterflies	*	*			*	*
Papilionidae: Swallowtails	*	*	*	*		
Pieridae: Sulfers	*	*	*			

Order Siphonaptera: Fleas					*	
Order Diptera: Flies						
Asilidae: Robber Flies	*			*		
Bibionidae: March Flies			*			
Bombyliidae: Bee Flies	*					
Calliphoridae, Blow Flies	*	*	*		*	
Chironomidae: Midges	*	*	*	*		*
Culicidae: Mosquitoes	*	*	*	*	*	*
Dolichopodidae: Long-legged Flies	*	*	*	*	*	*
Muscidae: House Flies	*	*	*	*	*	*
Mycetophilidae: Fungus Gnats	*					
Phoridae: Hump-backed Flies	*	*	*	*		*
Pipunculidae: Big-headed Flies	*	*	*	*		*
Sarcophagidae: Flesh Flies		*		*	*	*
Sepsidae: Scavenger Flies	*					
Simuliidae: Black Flies	*	*	*			
Stratiomyidae: Soldier Flies	*			*	*	*
Syrphidae: Flower Flies	*	*	*	*	*	*
Tabanidae: Horse Flies, Deer Flies	*	*	*			
Tachinidae: Tachinid Flies	*	*		*		
Tipulidae: Crane Flies	*	*	*	*		*
Ulidiidae: Picture-winged Flies	*	*	*	*	*	*
Taxonomic Richness	93	78	75	79	68	62

• Small Lepidoptera of the Super Families Gelechioidea, Pyraloidea, Tiniodea, Gracillarioidea, Incurvarioidea, and Families Tortricidae and Pterophoridae.

Table S6. Results of ANOVA and post-hoc Tukey HSD testing for differences in complexity for each of the three counties (Cass, Christian and Sangamon) at each of three scales (6000 m, 1000 m, and 500 m). Signif. codes: $p < 0.001$: '***'; >0.01: '*'; >0.05: '.' >0.1: NS.

a. Complexity 6000 m ~ County

Model	Df	Sum Sq	Mean Sq	F value	Pr(>F)	Signif.
County	2	1151	575.4	11.8	0.000208	***
Residuals	27	13.16	48.8			

Model	Diff	Lwr	Upr	P adj	Signif.
Cass-Sang	−2.2	−9.943	5.543	0.7629	NS
Chris-Sang	−14.1	−21.843	−6.353	0.0003	***
Chris-Cass	−11.9	−19.643	−4.153	0.0020	***

b. Complexity 1000 m ~ County

Model	Df	Sum Sq	Mean Sq	F value	Pr(>F)	Signif.
County	2	2087	1043	4.992	0.0143	*
Residuals	27	5643	209			

Model	Diff	Lwr	Upr	P adj	Signif.
Cass-Sang	3	−13.0297	19.0297	0.8885	NS
Chris-Sang	−16	−32.0297	0.0297	0.0504	.
Chris-Cass	−19	−35.0297	−2.9702	0.0177	*

c. Complexity 500 m ~ County

Model	Df	Sum Sq	Mean Sq	F value	Pr(>F)	Signif.
County	2	1176	588.2	1.845	0.177	NS
Residuals	27	8610	86.10			

Model	Diff	Lwr	Upr	P adj	Signif.
Cass-Sang	8.6	−11.2011	28.4011	0.5362	NS
Chris-Sang	−6.7	−26.5011	13.1011	0.6825	NS
Chris-Cass	−15.3	−35.1011	4.5011	0.1536	NS

Table S7. Models for testing the significance of county, Cass, Christian, or Sangamon County (a–b); year, 2011 or 2012 (c–d); and collection method (sticky board, pitfall trap, or sweep net (e–f) on taxonomic richness (TR) and diversity index (DI). Variables included Location (FI or FE); the proportion of non-agricultural area at the 1000 m scale, crop in the FI (soybeans or corn), closest adjacent field (soybeans, corn, grassland or developed), length of the FE (m), distance to nearest non-arable space > 1 ha (Green1ha), and a correction factor for sample size (ln Abundance). Our random factors were method of collection (sticky board, pitfall trap, or sweep net) and field within year, county within field or county within year within field. We included the identity of the sample (fID) to fit a quasi-Poisson distribution for TR.

	Models
a.	TR ~ Location * (County + Complexity1000 + Crop + Adjacent Field + Length + Green1ha) + ln Abundance + (1∣YearField)+(1∣Method) + (1∣fID)
b.	DI ~ Location * (County + Complexity1000 + Crop + Adjacent Field + Length) + ln Abundance + (1∣YearField) + (1∣Method)
c.	TR ~ Location * (Year + Complexity1000 + Crop + Adjacent Field + Length + Green1ha) + ln Abundance + (1∣CountyField) + (1∣Method) + (1∣fID)
d.	DI ~ Location * (Year + Complexity1000 + Crop + Adjacent Field + Length) + ln Abundance + (1∣CountyField) + (1∣Method)
e.	TR ~ Location * (Method + Complexity1000+ Crop + Adjacent Field + Length + Green1ha) + ln Abundance + (1∣CountyYearField) + (1∣fID)
f.	DI ~ Location * (Method + Complexity1000 + Crop + Adjacent Field + Length) + ln Abundance + (1∣CountyYearField)

© 2016 by the authors; licensee MDPI, Basel, Switzerland. This article is an open access article distributed under the terms and conditions of the Creative Commons by Attribution (CC-BY) license (http://creativecommons.org/licenses/by/4.0/).
